# Supplementary material for: Deletion of wfs1 Impairs Oligodendrocyte Precursor Cells Dorsal Distribution and Myelination Through the wfs1-hmgcs1 Axis in Zebrafish
Source: Biology (Basel). 2026 Aug 16;15(16):1402. doi: 10.3390/biology15161402 (PMC13510151; doi:10.3390/biology15161402)
Supplement: Supplementary file 1 [file biology-15-01402-s001.zip › File S1-Transcriptome sequencing data and differentially expressed genes (DEGs) in wfs1-- zebrafish at 5 dpf/2.KEGG/wfs1KOvsWT/DOWN/src/dre04510.html]

dre04510


- dre:797491

- Down regulated genes

797491\_1(-1.09418588609297)

- dre:563023

- Down regulated genes

563023(-1.03169619245437)

- dre:793158

- Down regulated genes

793158(-6.94943122774273)

- dre:30429

- Down regulated genes

30429\_2(-1.78714384880949)

- dre:100000252

- Down regulated genes

100000252\_1(-1.7718817168434)

- dre:556842

- Down regulated genes

556842\_1(-1.05551863440073)

- dre:497384

- Down regulated genes

497384\_1(-2.05267237809407)

- dre:436718

- Down regulated genes

436718\_1(-6.85571323128554)

- dre:373107

- Down regulated genes

373107\_1(-7.07514976036473)

- dre:58100

- Down regulated genes

58100\_1(-4.1286149197908)

- dre:386856

- Down regulated genes

386856\_1(-1.99487147218035) novel.946(-6.7676180476835)
- dre:571431

- Down regulated genes

571431(-6.42862549702529)
- dre:245702

- Down regulated genes

245702\_1(-1.45433245048201)
- dre:796537

- Down regulated genes

796537\_1(-2.50164719187441)

- dre:560564

- Down regulated genes

560564\_2(-4.02056550705436)
- dre:103909601

- Down regulated genes

378480(-1.33145438810737)

- dre:792699

- Down regulated genes

792699(-1.84389282152821)

- dre:57934

- Down regulated genes

57934(-1.25539807424297)

Close
